# Reversing NK cell exhaustion: a novel strategy combining immune checkpoint blockade with drug sensitivity enhancement in the treatment of hepatocellular carcinoma

**DOI:** 10.3389/fonc.2024.1502270

**Published:** 2025-01-21

**Authors:** Yuxiang Huang, Hengjian Liao, Jiefu Luo, Huaning Wei, Anling Li, Yujie Lu, Bangde Xiang

**Affiliations:** Department of Hepatobiliary Surgery, Guangxi Medical University Cancer Hospital, Nanning, China

**Keywords:** hepatocellular carcinoma, NK cell exhaustion, immunotherapy, immune checkpoint blockade, drug sensitivity, tumor microenvironment, oncology drug innovation

## Abstract

Hepatocellular carcinoma (HCC) is one of the most common lethal cancers worldwide. Natural killer cells (NK cells) play a key role in liver immunosurveillance, but in the tumor microenvironment, NK cells are readily depleted, as evidenced by down-regulation of activating receptors, reduced cytokine secretion, and attenuated killing function. The up-regulation of inhibitory receptors, such as PD-1, TIM-3, and LAG-3, further exacerbates the depletion of NK cells. Combined blockade strategies targeting these immunosuppressive mechanisms, such as the combination of PD-1 inhibitors with other inhibitory pathways (eg. TIM-3 and LAG-3), have shown potential to reverse NK cell exhaustion in preclinical studies. This article explores the promise of these innovative strategies in HCC immunotherapy, providing new therapeutic directions for optimizing NK cell function and improving drug sensitivity.

## 1 Introduction

Hepatocellular carcinoma (HCC) is one of the most common cancers in the world, accounting for about 90% of all primary liver cancer cases (1–3). Statistics show that HCC causes about 850,000 new cases and 800,000 deaths each year, and the extremely high mortality rate highlights a major threat to global public health (2, 4–6). HCC is often asymptomatic in the early stages, resulting in most patients being diagnosed at a later stage, with a poor prognosis and difficult to treat (7–10). The treatments for HCC include surgery, localized ablation, liver transplantation, chemotherapy, radiotherapy, and immunotherapy, etc (11–15). However, due to the complex structure of the liver and the heterogeneity of HCC, there are significant limitations in the practical application of these methods (16, 17). Immunotherapy, especially immune checkpoint inhibitors, is effective in some patients with HCC, but the overall response rate is low and drug resistance is easy to (18). Therefore, the development of new strategies that can augment the effect of the existing immunotherapeutic treatments is urgently needed.

**Figure 1:**
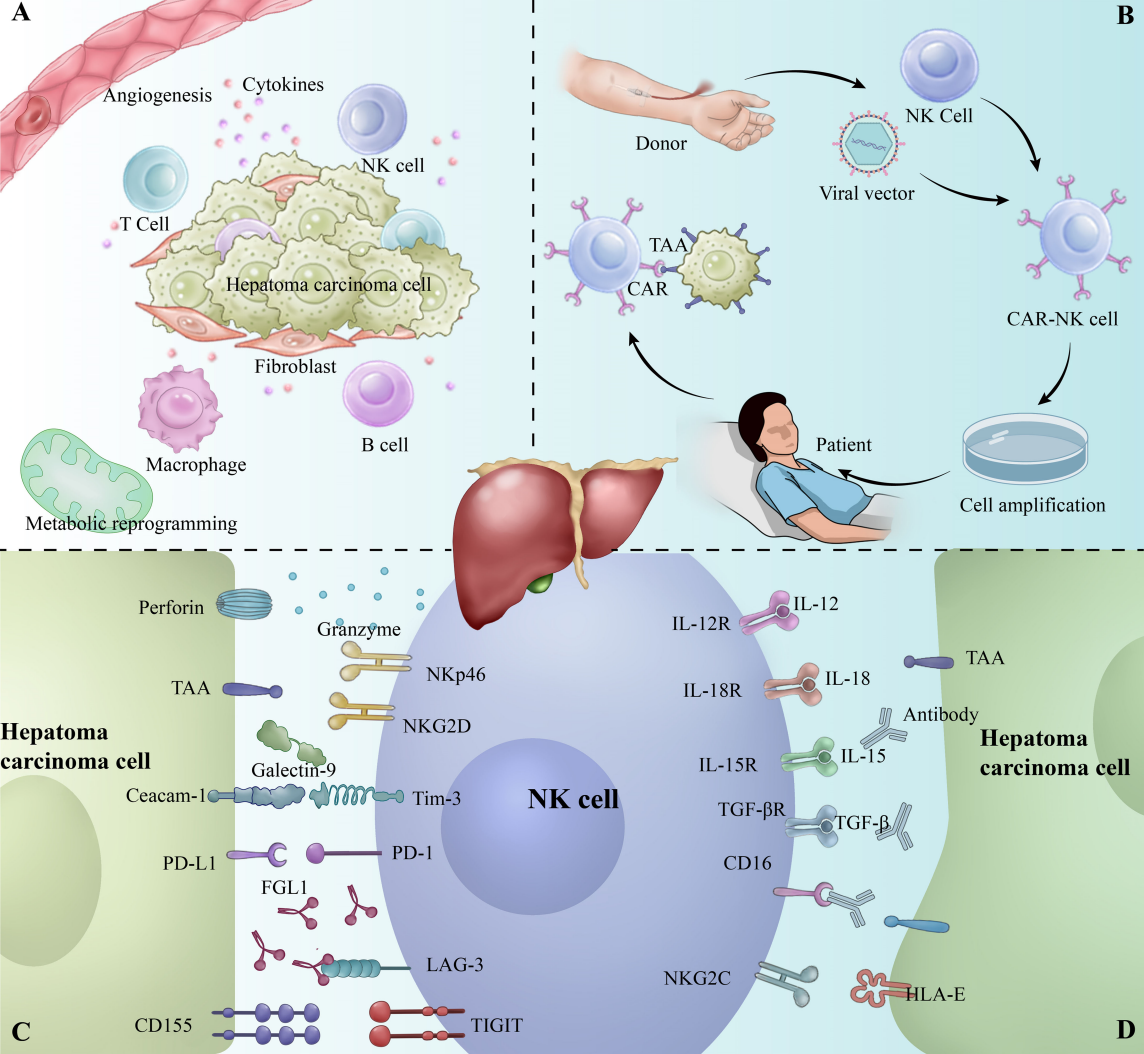
**(A)** Tumor microenvironment of hepatocellular carcinoma (HCC). **(B)** Workflow of CAR-NK therapy. **(C)** Overview of inhibitory receptors involved in the interaction between NK cells and tumor cells. **(D)** Overview of activating receptors involved in the interaction between NK cells and tumor cells.

Natural Killer Cells (NK cells) are an important component of the innate immune system, capable of rapidly recognizing and clearing virus-infected and tumor cells without relying on antigen presentation (19). In the hepatobiliary system of healthy adults, NK cells exhibit a unique behavioral pattern, with a high activity and frequency, occupying 22.6% of the total number of Intrahepatic lymphocytes (IHLs) (20). Studies have shown that NK cells are effective in inhibiting hepatocellular carcinoma development and progression in the liver microenvironment. However, immunosuppressive factors in the tumor microenvironment, such as TGF-β and IL-10, often impair the anti-tumor function of NK cells, leading to their functional depletion (19, 21). NK cell exhaustion refers to the gradual loss of NK cell function due to prolonged exposure to tumor antigens and inhibitory signals in the tumor microenvironment, and is characterized by downregulation of activation receptors, decreased cytokine secretion, and decreased killing capacity. It is also accompanied by upregulation of inhibitory receptors such as PD-1, TIM-3, and LAG-3 (**Figure 1C**) (22–24). These changes enable tumor cells to evade immune surveillance and promote growth and metastasis.

Understanding for these mechanisms of immunosuppression provides new directions for the optimization of immunotherapy. By combined blockade of multiple inhibitory pathways such as PD-1, TIM-3, LAG-3, and others, researchers are working to reverse NK cell exhaustion (25, 26). Emerging therapies such as immune checkpoint inhibitors, chimeric antigen receptor for NK cells (CAR-NK) therapies, and immunomodulatory drugs (e.g., IL-15 and IL-2) show great potential to enhance the antitumor activity of NK cells and improve drug sensitivity (27–30).

## 2 Biology of NK cells

### 2.1 Generation, differentiation and function of NK cells

NK cells (natural killer cells) are important effector cells of the immune system responsible for anti-tumor and anti-viral resistance, derived from bone marrow hematopoietic stem cells and matured through a multi-step differentiation process (31, 32). Human NK cells are classified into two major subpopulations based on the expression of CD16 and CD56. The majority (85%-95%) of NK cells in peripheral blood are of the CD56-CD16+ subpopulation, displaying a developmentally mature phenotype and possessing a high degree of cytotoxicity (33, 34). Unlike T cells, which depend on antigen presentation, NK cells are capable of lysing without the need for antigen presentation, and are able to pass through lysates containing granzyme B and perforin. Cell particles containing granzyme B and perforin cross the immune synapse to mediate sequential killing of infected or malignant cells (35).

In contrast, the CD56+CD16- subpopulation has fewer NK cells and exhibits an immature phenotype. Although the latter have low cytotoxicity when not activated, they are capable of producing large amounts of cytokines and exerting potent immunomodulatory effects when stimulated by pro-inflammatory cytokines (e.g., IL-15) (36, 37). The two subpopulations complement each other functionally, with the former being primarily responsible for the direct killing effect, and the latter playing an important role in immune response via cytokine secretion.

Upon maturation, NK cells have a potent killing capacity, although this cytotoxicity is not associated with major histocompatibility complex (MHC) expression because NK cells do not express somatically rearranged antigen receptors (38, 39). Instead, NK cells regulate NK cell activity through a balance of their activating (e.g., NKG2D, NKp46) and inhibitory (e.g., KIR family) receptors, thereby killing or causing tolerance in target cells (40, 41).

CD16 (low affinity IgG Fc region receptor III, FcγRIII), the most potent activating receptor expressed by NK cells and the only receptor that can activate NK cells on its own, can assist antibody-mediated immune responses through the antibody-dependent cell-mediated cytotoxicity (ADCC) pathway (42–44). The Fc portion of the antibody was engineered to increase its affinity for CD16a and enhance the ADCC effect. For example, replacing four amino acids in the Fc region to become a GASDALIE mutant significantly enhanced the affinity of Fc for CD16a, while the affinity for CD32b remained almost unchanged (45).

NKG2D protein is an important activating cell surface receptor protein, which is mainly expressed on cytotoxic immune cells, such as NK cells, CD8+ T cells, etc (46, 47). The first known NKG2D receptor is MICA with MICB, which is expressed in a wide variety of tumors (liver cancer, breast cancer, lung cancer) and organ transplant recipient MIC is expressed in tissue cells (48–51). MICA exhibits a very low tumor mutation burden, suggesting that its expression is not significantly affected by DNA editing mechanisms, so NKG2DL overexpression may be a potent strategy for anti-tumor progression (52, 53).

### 2.2 NK cells in the liver

NK cells are distributed in high concentrations in the liver, accounting for 50% of hepatic innate immune cells, making the liver one of the major sites of NK cell residency (31, 54, 55). This feature arises from the fact that embryonic hepatic hematopoietic stem cells are divided into two fractions, one of which continues to remain in the adult liver, generating the characteristic tissue-resident NK cells (LrNK) (56).

Compared to peripheral blood, NK cells in the liver differ in both effector molecule expression and cellular activity. The CD56+CD16- subpopulation is predominant in the liver (33). LrNK cells have a suppressive function in the immune tolerance microenvironment of the liver, particularly in inhibiting the antiviral response of T cells through the PD-1/PD-L1 pathway. For example, Zhou et al. found that exogenous transfusion of LrNK cells to normal or LrNK cell-deficient mice suppressed antiviral T-cell responses in the liver and was dependent on the PD-1-PD-L1 axis. In contrast, NK (cNK) cells circulating in the peripheral blood promoted T-cell responses (57).

## 3 Immunosuppressive factors and NK cell exhaustion mechanisms in the HCC tumor microenvironment

The tumor microenvironment (TME) of HCC consists of immune cells, immunosuppressive cells, and mesenchymal stromal cells with hypoxia, angiogenesis, metabolic reprogramming, inflammation, and immunosuppression (**Figure 1A**) (58, 59). The TME of HCC is characterized by the secretion of a variety of immunosuppressive factors, such as interleukin (IL)-6, IL-10, and transforming growth factor-β (TGF-β), prostaglandin E2 (PGE2) to directly inhibit NK cell activity and promote NK cell exhaustion (60–64).

TGF-β is known to be a potent immunosuppressive factor, which impairs tumor cell recognition by NK cells by down-regulating the expression of activation receptors on the surface of NK cells, such as NKG2D (65, 66). In addition, TGF-β further impairs its anti-tumor effect by inhibiting IFN-γ production and ADCC in NK cells (67, 68). IL-10, on the other hand, impairs its killing activity by inhibiting NK cell proliferation and cytokine secretion (e.g., IFN-γ and TNF-α) (69).

In addition, IL-6, as another important immunosuppressive factor, further promotes immunosuppression in the tumor microenvironment through a complex signaling mechanism. Studies have shown that in intrahepatic cholangiocarcinoma (ICC) cells, IL-6 induces the expression of cyclic RNA (circRNA) GGNBP2 (cGGNBP2). cGGNBP2 encodes a protein, cGGNBP2-184aa, which forms a positive feedback loop that sustainably activates the STAT3 signaling pathway, thereby promoting tumor cell proliferation and metastasis (70). This sustained STAT3 activation indirectly inhibits NK cell function by regulating other immune cells in the tumor microenvironment, further promoting NK cell exhaustion.

These inhibitory factors not only act directly on NK cells, but also indirectly promote NK cell exhaustion by modulating the function of other immune cells such as regulatory T cells (Tregs) and myeloid-derived suppressor cells (71, 72). For example, this multilayered inhibitory mechanism causes NK cells to gradually lose their function in the TME, which facilitates immune escape from the tumor.

In the TME of HCC, the complex interaction of NK cells with other immune cells forms a suppressive network that further exacerbates NK cell functional exhaustion. Immunosuppressive cells such as dendritic cells (DCs) and regulatory T cells further diminish NK cell activity through secretion of inhibitory factors or direct cell-to-cell contact (73). DCs often exhibit abnormal function in HCC, which are unable to efficiently activate NK cells, but instead may further inhibit NK cell activity through the high expression of PD-L1 on the surface (74). Meanwhile, the increase of Tregs is also one of the important reasons for the suppression of NK cell function in TME. Tregs directly inhibit the activation and function of NK cells through cellular indirect contact and secretion of TGF-β and IL-10 (75).

## 4 Mechanisms of activation of exhaustion signaling pathways

NK cell exhaustion is closely associated with the activation of specific signaling pathways, of which the PD-1/PD-L1 pathway is one of the most important (**Figure 1D**). PD-1 is an inhibitory receptor that is highly expressed in the depleted state of NK cells, whereas its ligand, PD-L1, also exhibits a significant up-regulation in tumor cells and tumor-associated immune cells (76). When PD-1 binds to PD-L1, it inhibits the killing function and cytokine secretion of NK cells by down-regulating the activation signals in NK cells (77, 78). In addition, the PD-1/PD-L1 signaling pathway inhibits the activation of the Akt and mTOR pathways in NK cells, leading to metabolic dysfunction and further weakening its anti-tumor effects (79).

However, PD-1/PD-L1 is not the only signaling pathway driving NK cell exhaustion. Other inhibitory receptors such as TIM, TIGIT and LAG-3 are likewise significantly up-regulated in NK cells in the depleted state, forming a co-inhibitory network (26, 80).

### 4.1 TIM-3

TIM-3 is highly expressed in the depleted state of NK cells and acts as a co-inhibitory receptor involved in the modulation of type I immune responses. The immunomodulatory mechanism of TIM-3 is dependent on its binding to several ligands, such as Galectin-9, phosphatidylserine (PtdSer), HMGB1 and CEACAM-1 (81). PtdSer acts as an “eat-me” signal that promotes the clearance of apoptotic cells by binding to TIM-3. HMGB1, a damage-associated molecular pattern (DAMP), regulates the innate immune response by suppressing the inflammatory response when it binds to TIM-3. Binding of CEACAM-1 is thought to be closely related to inhibitory signaling by TIM-3.

These ligands, including CEACAM-1, Galectin-9, PtdSer, and HMGB1, bind to different regions of TIM-3, respectively, triggering intracellular inhibitory signals (82). For example, upon binding of TIM-3 to Galectin-9, the Y256 and Y263 sites in its cytoplasmic domain are phosphorylated, leading to dissociation of the articulator BAT3 from TIM-3, which in turn inhibits TCR signaling and reduces NK cell immune response, especially reducing the secretion of key cytokines such as IFN-γ (82). Phosphorylation of the Y256 and Y263 sites is not only a critical step in TIM-3-regulated signaling, but also promotes the activation of other inhibitory signals by preventing the binding of BAT3 to TIM-3.

### 4.2 TIGIT

The co-inhibitory receptor, TIGIT, blocks the direct interaction of NK cells with tumor cells by binding to the ligands CD155 and CD112, which are highly expressed in antigen-presenting cells (APCs) and tumor cells, diminishing their killing ability and further inhibiting cytokine secretion by NK cells, such as TNF-α and IFN-γ (83, 84). In addition, TIGIT interferes with tumor recognition by NK cells through competitive inhibition of CD226. Although TIGIT shares the same ligand as CD226, it binds to CD155 and CD112 with higher affinity, thereby inhibiting CD226-mediated activation signaling. This competitive mechanism further exacerbates the depleted state of NK cells (84).

### 4.3 LAG-3

LAG-3 is a structurally similar inhibitory receptor to CD4 that inhibits NK cell activation mainly through binding to MHC class II molecules (85). LAG-3 is highly expressed not only in T cells but also upregulated on NK cells, and this upregulation leads to suppression of both innate and adaptive immune functions in tumor patients.

LAG-3 hinders effective recognition and clearance of tumor cells by NK cells through interaction with FGL1 (fibrinogen-like protein), which is highly expressed in hepatocytes and tumor cells. In addition, LAG-3 regulates downstream molecules (e.g., SHP-1 and SHP-2) through inhibitory signaling motifs (e.g., FXXL motifs and KIEELE motifs) in its intracellular structural domains, further blocking the activation signaling pathway of NK cells by dephosphorylating activating signaling molecules (26, 86).

Co-upregulation of these inhibitory receptors is particularly evident in response to chronic antigenic stimulation, especially in NKG2C+ NK cells, where the expression of LAG-3 and PD-1 rises progressively over time (87).

## 5 Immunotherapy and drug sensitization in the restoration of NK cell function

### 5.1 Immune checkpoint inhibitors (PD-1/PD-L1)

Immune checkpoint inhibitors (ICIs), especially PD-1/PD-L1 inhibitors, have demonstrated significant clinical efficacy in the treatment of a variety of solid tumors, such as lung, breast, advanced hepatocellular, and pancreatic cancers (6, 88–92). These inhibitors work by blocking the binding of PD-1 to its ligand PD-L1, restoring the anti-tumor activity of NK cells and T cells, and increasing their cytotoxicity and secretion of immune factors such as IFN-γ and TNF-α. In different types of tumors, including HCC, lung cancer, and melanoma, NK cells have shown variable responses to PD-1/PD-L1 inhibitors, influenced by the tumor microenvironment and the extent of PD-1 expression on NK cells. It has been found that not all of the PD-1 in NK cells is derived from endogenous expression, and that NK cells also acquire PD-1 and other inhibitory substances from the membrane of the tumor cells through SLAM receptor-mediated trogocytosis. NK cells can also acquire inhibitory molecules such as PD-1 from the membrane of tumor cells through SLAM receptor-mediated trogocytosis (93). This process results in the suppression of the anti-tumor function of NK cells, which can be reversed by PD-1 inhibitors.

### 5.2 Joint innovative applications of immunotherapy

Although PD-1/PD-L1 inhibitors show good single-agent efficacy in some tumors, single-agent efficacy is typically lower in metastatic tumors of the hepatobiliary system, and patients experience initial resistance or subsequent decreased drug sensitivity. Therefore, investigators are exploring further enhancement of the anti-tumor effects of NK cells through combination therapies (6, 94–96). The combined blockade of PD-1/PD-L1 inhibitors with other inhibitory pathways has shown promising potential in reversing immune exhaustion during hepatocellular carcinoma treatment. TIM-3, is highly expressed in the TME of HCC, especially on tumor-infiltrating NK cells (e.g., cNK and LrNK cells). Studies have shown that TIM-3, through binding to its ligand phosphatidylserine (PtdSer), induces inhibition of downstream signaling pathways such as PI3K/mTOR, which in turn leads to dysregulation of NK cells and tumor evasion of the immune (6). Through gene ablation, antibody blockade, or lentiviral-mediated TIM-3 disruption experiments, the researchers succeeded in restoring NK cells’ cytokine secretion (e.g., IFN-γ, TNF-α) and cytotoxicity, significantly inhibiting HCC growth (88).

IL-15 and IL-2, two of the most widely studied cytokines, are able to enhance anti-tumor efficacy by stimulating NK cell proliferation and enhancing their effector functions. IL-15 significantly increases NK cell cytotoxicity by activating the downstream JAK/STAT signaling pathway through binding to IL-15Rα (97, 98). IL-2 is also capable of enhancing NK cell activation through the CD25 receptor, but is less used due to its tendency to induce proliferation of Tregs. Currently, researchers are developing modified versions of IL-2, such as mutant IL-2 with selective activation of NK cells, to avoid the side effects of Tregs (99).

## 6 Conclusion and future

With the development of technologies such as single-cell sequencing, we have the opportunity to further explore the TME and reveal the complex immune cell interactions therein. Although the presence of NK cells in the TME has long been recognized, it remains challenging to effectively manipulate NK cells for therapeutic purposes. NK cell exhaustion in HCC is closely associated with the upregulation of immune checkpoints. Strategies to restore NK cell function, such as immune checkpoint inhibition and cytokine therapy, have shown promise in clinical studies. Notably, CAR-NK cell therapy, with its broad anti-tumor activity and low immune rejection (**Figure 1B**) (102). It has demonstrated success in hematologic cancers and offers new hope for treating solid tumors like HCC (27, 100, 101). Moving forward, the combination of CAR-NK therapy with other immunotherapies, along with advances in single-cell technologies, will drive further progress in HCC immunotherapy.
